# Mechanistic study of quercetin on *Fagopyrum Tataricum* resistant starch

**DOI:** 10.1016/j.fochx.2026.103883

**Published:** 2026-04-20

**Authors:** Shengchun Li, Haixia Zhong, Anhu Wang, Qiuping Wei, Lan Wang, Wenhui Ma, Zhiguang Chen

**Affiliations:** a Xichang University, College of Agricultural Sciences, Panxi Crop Improvement Key Laboratory of Sichuan Province, Xichang, Sichuan Province 615000, China; b School of Basic Medical Sciences, Department of Biochemistry, Hubei University of Medicine, Shiyan 442000, China

**Keywords:** Resistant starch, Quercetin, *Fagopyrum tataricum* starch

## Abstract

In this study, the interaction between quercetin and *Fagopyrum tataricum* starch, as well as the mechanism by which it increases the RS content, was analyzed. The results showed: (1) at 5% addition, RS content reached 60.38%. (2) Quercetin penetrated starch granules and blocked pore structures, reducing the accessible surface area and hindering amylase hydrolysis: the first mechanism for RS increase. (3) The internal quercetin formed stable complexes with starch molecules (90 kJ/mol binding energy, 7H-bonds), reconstructing the hydrogen bonding network, altering molecular arrangement, and modifying gelatinization properties. Due to steric hindrance, the starch molecule complexed with quercetin failed to fully insert into the α-amylase active groove, limiting contact with the catalytic triad: the second mechanism for RS increase. (4) External quercetin molecules bound stably within the α-amylase active groove (49 kJ/mol binding energy, 5H-bonds), inhibiting enzyme activity: the third mechanism for RS increase.

## 1 Introduction

Diabetes currently represents one of the most significant chronic diseases threatening human health globally. According to the International Diabetes Federation (IDF), approximately 590 million people worldwide are living with diabetes of varying severity (Młynarska et al., 2025). Resistant starch (RS), defined as the fraction of starch that escapes enzymatic digestion in the small intestine of healthy individuals within 120 min of ingestion, plays a critical role in the clinical management of diabetes (Kovacs et al., 2025).

Currently, resistant starch is classified into five types: RS1 refers to naturally physically entrapped starch; RS2 refers to native resistant starch granules; RS3 refers to resistant starch formed through retrogradation; RS4 refers to resistant starch prepared by chemical modification; and RS5 refers to resistant starch formed by complexation of starch with small molecules.

The primary mechanism of RS5 involves the complexation of starch with other small molecules, which generates steric hindrance and consequently reduces the rate of starch hydrolysis. Researchers have primarily prepared RS5 by adding lipids to starch. For example, Esquivel-Fajardo et al. added oleic acid to waxy corn starch, increasing its RS content from 20.83% to 49.33% (Esquivel-Fajardo et al., 2024). Similarly, Li et al. incorporated lauric acid into *Canna edulis* starch, raising its RS content to 56.12% (Li, Zhang, Wang, Liu, et al., 2025). Compared with lipids, polyphenols are more desirable raw materials for preparing type 5 RS. On one hand, polyphenols possess multiple hydroxyl groups, which enable higher complexation efficiency than lipids. On the other hand, different polyphenols exhibit various physiological functions, such as antioxidant, lipid-lowering, and gut microbiota-regulating activities (Lee et al., 2013; Luthar et al., 2020). In recent years, many researchers have attempted to use polyphenols for RS5 preparation. For instance, Li et al. added baicalein and puerarin to corn starch, increasing the RS content by 103.95% and 271.36%, respectively (Li, Wang, Wu, Tan, et al., 2025). In other studies, the addition of tea polyphenols and ferulic acid to yam starch raised its RS content to 56.25% and 54.15%, respectively (Xie et al., 2023; Zhang et al., 2023).

*Fagopyrum tataricum*, often hailed as the “king of coarse grains”, has high nutritional value, being rich in flavonoids, essential amino acids, and high-value unsaturated fatty acids. Before processing, *Fagopyrum tataricum* must undergo dehulling. The removed hulls account for approximately 24% of the total grain weight and are usually discarded directly, resulting in a serious waste of resources (Li, Yu, Guo, Yan, & Jiang, 2025). Preliminary experiments in our study revealed that the total flavonoid content in *Fagopyrum tataricum* hulls is 9.87 mg/g, of which about 96% is quercetin.

Numerous studies have confirmed that quercetin possesses multiple biological activities, including hypoglycemic, hypolipidemic, antihypertensive, antioxidant, and gut microbiota-regulating effects (Cui et al., 2019; Dzah et al., 2020; Peng et al., 2019; Shen et al., 2025; Wang et al., 2022, Wang, Liu, Liu, Guo and Li, 2022; Zhou et al., 2025; Zielińska et al., 2012). Therefore, adding quercetin to *Fagopyrum tataricum* products can significantly enhance their nutritional value. Moreover, the quercetin molecule contains six hydroxyl groups, indicating strong hydrogen bonding interactions with starch molecules, making it an ideal material for RS5 preparation.

If quercetin can be extracted from *Fagopyrum tataricum* hulls and added back to *Fagopyrum tataricum* flour, it would not only increase the RS content and nutritional value of *Fagopyrum tataricum* but also turn the processing by-product into a valuable resource (Fig. S1). Therefore, in this study, the interaction between quercetin and *Fagopyrum tataricum* starch, as well as the mechanism by which it increases the RS content, was analyzed. The results will provide important references for the preparation of RS5 and lay a crucial foundation for the recycling of discarded *Fagopyrum tataricum* hull resources.

## 2 Materials and methods

### 2.1 Extraction of quercetin and its incubation with *Fagopyrum tataricum* starch

The *Fagopyrum tataricum* material used in this study was Xiqiao 2, a variety with a high RS content (35.03%) obtained by our team through the screening and optimization of cultivation conditions. The dried *Fagopyrum tataricum* hulls were ground into powder and subjected to ultrasound-assisted extraction using ethanol as the solvent to enhance extraction efficiency. The resulting extract was filtered, concentrated, and subsequently purified using macroporous adsorption resin to further enrich the flavonoid fraction. Acid hydrolysis was then employed to convert rutin in the extract into quercetin aglycone. (Note: Due to the large quantity of quercetin required in this study, analytical-grade quercetin was used in the incubation experiments to ensure efficiency and accuracy.)

Quercetin was added to *Fagopyrum tataricum* starch at concentrations of 1%, 2.5%, 5%, 7.5%, and 10% (*w*/w), with a starch suspension concentration of 20% (higher concentrations led to starch sedimentation). The mixed suspensions were incubated on a shaker at 200 rpm for 3 h at room temperature. Water-treated (without quercetin) *Fagopyrum tataricum* starch was used as the control. After incubation, the samples were centrifuged to remove excess water, freeze-dried, and subjected to analyses including RS content determination, SEM, 3D-CT, FTIR, XRD and low-temperature nitrogen adsorption measurements.

### 2.2 Determination of RS content

Resistant starch (RS) content in *Fagopyrum tataricum* samples was determined using the K-RSTAR Resistant Starch Assay Kit (Megazyme, Ireland) following the manufacturer's instructions. Briefly, samples were incubated with pancreatic α-amylase and amyloglucosidase in a shaking water bath at 37 °C for 16 h to hydrolyze digestible starch. The reaction was terminated by the addition of ethanol, followed by centrifugation to remove the supernatant containing the digested starch fraction. The pellet was washed with 50% ethanol and subsequently treated with 2 M KOH in an ice bath to dissolve the resistant starch. After neutralization with acetate buffer, the resistant starch was hydrolyzed to glucose by amyloglucosidase. Glucose content was quantified colorimetrically at 510 nm using glucose oxidase/peroxidase reagent (GOPOD), and the RS content was calculated accordingly.

The hydrolysis rate was determined using the K-DSTRS kit. Samples were incubated with amylase and amyloglucosidase in pH 6.0 maleate buffer at 37 °C with shaking. At 20, 40, 60, 80, 100, and 120 min, 1.0 mL aliquots were withdrawn, and the reaction was stopped by adding 50 mM acetic acid. After secondary hydrolysis with glucoamylase, the glucose content was measured using the GOPOD reagent, and the hydrolysis rate was calculated based on the amount of glucose released.

Each experiment was performed in triplicate. Significance analysis was conducted using SPSS software, employing the *t*-test and the least significant difference (LSD) method for multiple comparisons.

### 2.3 Scanning electron microscopy (SEM)

The prepared *Fagopyrum tataricum* starch samples were ground, dispersed, and passed through a 100-mesh sieve. Approximately 100 mg of the sample was placed into a 2 mL screw-cap tube. Subsequently, 1 mL of 4% SDS-wash buffer was added, and the mixture was vortexed thoroughly, followed by centrifugation at 10,000 rpm for 1 min. The supernatant was discarded, and this washing step was repeated twice. The pellet was then washed with 1 mL of deionized water, vortexed, centrifuged at 10,000 rpm for 1 min, and the supernatant was discarded; this water wash was repeated five times. Finally, 1 mL of anhydrous ethanol was added and mixed by vortexing. A small volume (approximately 200 μL) of the prepared starch suspension was pipetted onto conductive adhesive tape attached to a copper stub, spread evenly, and left to dry overnight at 37 °C. The samples were sputter-coated with gold prior to observation.

The granular morphology of *Fagopyrum tataricum* starch was observed using a high-resolution field emission scanning electron microscope (Zeiss Merlin Compact, Carl Zeiss, Japan). The instrument was equipped with a thermal field emission Schottky electron gun (accelerating voltage: 0.02–30 kV; beam current: 12 pA–100 nA; beam stability: <0.2%/h). Both the in-lens Duo secondary electron detector and the Everhart–Thornley secondary electron detector were utilized for imaging.

### 2.4 Low-temperature nitrogen adsorption

The pore structure parameters of starch samples were determined by low-temperature nitrogen adsorption at 77 K using a specific surface area and porosity analyzer. Prior to measurement, each sample (approximately 0.1–0.3 g) was degassed under vacuum at 80 °C for 10 h to remove adsorbed moisture and volatiles. The Brunauer-Emmett-Teller (BET) method was applied to calculate the specific surface area (SBET), and the Barrett-Joyner-Halenda (BJH) model was used to determine pore size distribution and average pore diameter based on the desorption branch of the isotherm. The statistical analysis method was the same as that used for RS testing.

### 2.5 X-ray diffraction (XRD)

XRD characterization was performed according to the method described by Wang et al. (Wang et al., 2025). Starch samples equilibrated for moisture content were ground in a mortar, dispersed, and passed through a 100-mesh sieve. An appropriate amount of sample (approximately 100 mg) was loaded onto a sample holder, pressed flat, and evenly distributed. X-ray diffraction patterns were recorded using an X-ray diffractometer equipped with a copper target (Cu Kα radiation, λ = 0.15406 nm) operating at 1600 W (40 kV × 40 mA). The X-ray intensity was measured using a NaI crystal scintillation counter. Diffractograms were acquired over a 2θ range of 4° to 60° with a step size of 0.02° and a scanning rate of 4°/min. The divergence slit (DS), scattering slit (SS), and receiving slit (RS) were set to 0.5 mm, 0.25 mm, and 0.1 mm, respectively.

### 2.6 Differential scanning calorimetry (DSC) analysis

DSC characterization was performed according to the method described by Wang et al. (Wang et al., 2025). The prepared *Fagopyrum tataricum* starch samples were ground, dispersed, and sieved through a 100-mesh screen. An accurately weighed sample (2.5–3.1 mg) was placed into a DSC pan, and an appropriate amount of deionized water was added. The pan was hermetically sealed and allowed to equilibrate at 4 °C for 24 h. Thermal analysis was performed using a differential scanning calorimeter (TA Instruments Q2000, TA Instruments, USA). The instrument detects phase transitions by measuring heat flow changes associated with thermal events. The scanning conditions were as follows: samples were heated from 30 °C to 105 °C at a rate of 10 °C/min.

Data analysis was conducted using Universal Analysis or Proteus Thermal Analysis software. The To, Tp, Tc and gelatinization enthalpy (ΔH) of the phase transition were calculated to characterize the thermal properties of the samples.

### 2.7 Fourier transform infrared (FTIR) spectroscopy

FTIR characterization was performed according to the method described by Yan et al. (Yan et al., 2024). *Fagopyrum tataricum* starch samples were ground, dispersed, and passed through a 100-mesh sieve. A small amount of the prepared sample (approximately 50 mg) was mixed with KBr and pressed into a pellet. FTIR spectra were recorded using a Fourier transform infrared spectrometer (Nicolet iZ-10, Thermo Fisher Scientific, USA). Spectra were acquired over a wavenumber range of 4000 to 400 cm^−1^ at a resolution of 4.00 cm^−1^, with 32 scans co-added. The measurement parameters included a gain of 8.0, mirror velocity of 0.4747 cm/s, aperture setting of 80.00, DTGS KBr detector, and KBr beam splitter.

### 2.8 High-resolution 3D-CT

The 3D-CT characterization referenced our previous study (Li et al., 2026). The internal structure of *Fagopyrum tataricum* starch granules was analyzed using a high-resolution 3D X-ray microscope (Voxel-3000, Sanying Precision Instruments Co., Ltd., China). Scanning was performed at an accelerating voltage of 60 kV and a current of 15 μA. A total of 1440 projection frames were acquired over a total scan time of 36 h. Each starch sample was scanned in triplicate, yielding three-dimensional grayscale matrix data with dimensions of 2500 × 2500 × 2100 voxels.

### 2.9 Molecular docking and simulation

The molecular docking and simulation referenced the method described in the study by Zhong et al. (Zhong, Yang, et al., 2024). As illustrated in Fig. S2, the three-dimensional structure model of human α-amylase was downloaded from the RCSB Protein Data Bank (PDB). The quercetin model was constructed using ChemDraw, and the starch molecular model was built using Amber tools.

Molecular docking of the starch–quercetin complex with α-amylase was performed using Discovery Studio software. The receptor was prepared with CHARMM36 force field at pH 7.4, and quercetin was minimized with CGenFF. The active site was defined as a 1–1.2 nm sphere around the catalytic center. 200 poses were generated with 1000 orientations, followed by post-docking minimization. The best conformation was selected based on CDOCKER interaction energy and hydrogen bond formation. The docking results were visualized using VMD (Visual Molecular Dynamics) software.

Subsequently, molecular dynamics (MD) simulations were performed on the models obtained from docking, with three parallel simulations conducted for each model. The simulation parameters were set according to our previous study (Zhong, Yang, et al., 2024): temperature (25 °C) and pressure (0.1 MPa) were controlled using the Langevin thermostat and Berendsen barostat, respectively. All simulations were carried out using Amber tools under the NPT ensemble for 100 ns, preceded by a 2 ns NVT equilibration and a 2 ns NPT equilibration. A cutoff distance of 1 nm was applied, and periodic boundary conditions along with the leapfrog integration algorithm were employed.

Simulation snapshots were extracted every 0.01 ns to calculate the following parameters: radius of gyration (Rg), root mean square fluctuation (RMSF), root mean square deviation (RMSD), solvent-accessible surface area (SASA), binding free energy, number of hydrogen bonds, and interatomic distances. Binding free energy was calculated using the MM-PBSA (Molecular Mechanics/Poisson–Boltzmann Surface Area) method. SASA was determined using the pairwise linear combination of overlapping spheres algorithm defined by Weiser. Hydrogen bonds were identified based on a distance criterion of <0.35 nm.

## 3 Results and discussion

### 3.1 Resistant starch content

As shown in Fig. S3, the RS content in native *Fagopyrum tataricum* starch was approximately 35%. With increasing quercetin supplementation, the RS content progressively increased. When quercetin was added at 5%, the RS content was significantly elevated to 60.38% (*P* < 0.01). In addition to quercetin, other polyphenols such as tea polyphenols, baicalein, puerarin, and ferulic acid have also been used for the preparation of RS5. Studies have shown that baicalein and puerarin increased the RS content of corn starch by 103.95% and 271.36%, respectively (Li, Wang, Wu, Tan, et al., 2025), while tea polyphenols and ferulic acid increased the RS content of yam starch to 56.25% and 54.15%, respectively (Xie et al., 2023; Zhang et al., 2023). However, further increases in quercetin concentration (7.5% and 10%) resulted in only marginal additional increases in RS content (63.44% and 64.13%, respectively), with no significant differences among these groups (*P* > 0.05). Therefore, a 5% quercetin addition level was considered optimal for practical applications. Accordingly, this concentration was selected for subsequent analyses, including SEM, 3D CT, XRD, and DSC. Due to the bitter taste of quercetin, processing methods with high moisture content (e.g., boiling) should be avoided in practical applications to prevent the release of free quercetin. Alternatively, additives may be used to improve the sensory properties of the product (Wan et al., 2024).

In recent years, researchers have also prepared (or discovered) several high-RS starches using various methods. Examples include corn starch (RS5) with an RS content of 80.78% prepared by adding butyric acid (Li et al., 2020); potato starch with an RS content of 83.21% after dry heat treatment (Guo et al., 2023); high-pressure treated rice starch with an RS content of 64.0% (Chan et al., 2019); native high-amylose corn starch with an RS content of 66% (Nagahata et al., 2013); and native banana starch with an RS content of 74.1% (Zhang et al., 2021).

RS content of starch is modulated by multiple structural factors across different hierarchical scales: (1) higher amylose content restricts granule swelling, impeding enzyme penetration and thereby increasing RS content; (2) higher molecular weight or larger radius of gyration enhances granule swelling, facilitating enzyme penetration and thus reducing RS content; (3) a higher proportion of A chains (DP6–12) diminishes double helix formation, thereby enhancing catalytic efficiency and decreasing RS content; (4) higher crystallinity or gelatinization enthalpy reflects a greater abundance of double helices, which hinders enzyme recognition and correlates with increased RS content; and (5) larger specific surface area provides a greater contact area for enzymatic action, consequently lowering RS content.

### 3.2 SEM and low-temperature nitrogen adsorption

**Fig. 1:**
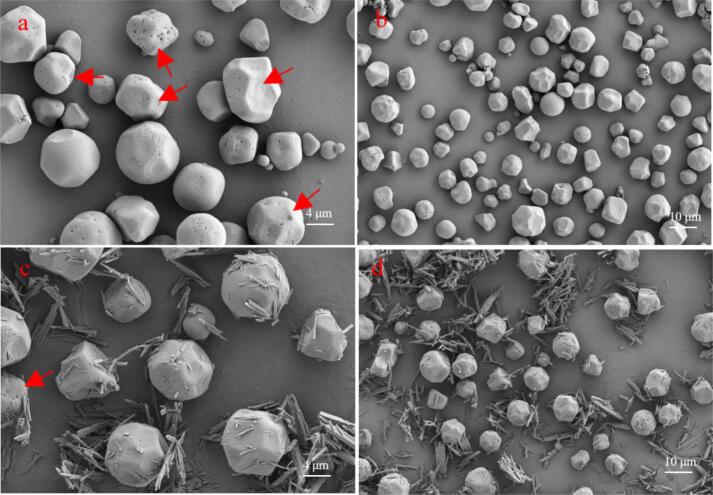
SEM imaging of *Fagopyrum tataricum* starch-quercetin.

As shown in Fig. 1, the morphological changes of *Fagopyrum tataricum* starch granules following interaction with quercetin (at a 5% concentration) were observed by scanning electron microscopy. Firstly, quercetin treatment exhibited no significant effect on the particle size or overall morphology of the starch granules, which retained their characteristic angled (truncated) features. Importantly, numerous pore structures were evident on the surface of native *Fagopyrum tataricum* starch granules (indicated by red arrows). Studies suggest that these pores may connect the granule surface to its interior, allowing enzymes to penetrate directly into the granule for hydrolysis, and are thus closely associated with starch digestibility (Bae et al., 2020; Vahidi et al., 2019). Following incubation with 5% quercetin, a noticeable reduction in the number of surface pores was observed. This may be attributed to the infiltration of quercetin molecules into the pores during incubation, effectively blocking these channel structures. This pore-blocking effect may be a significant contributing factor to the increased RS content in *Fagopyrum tataricum* after quercetin incubation.

It is worth noting that the molecular weight of quercetin is 302, and calculations based on its molecular structure yield a radius of gyration of approximately 1.5 nm. From Fig. 1a, it can be estimated that the pore diameters on the *Fagopyrum tataricum* starch granules are at least 500 nm to 1 μm. Furthermore, each quercetin molecule possesses six hydroxyl groups, which readily form hydrogen bonds with the hydroxyl groups of starch molecules, leading to their entrapment within the starch matrix. Therefore, at higher concentrations, quercetin molecules may fully penetrate and block the pore structures of *Fagopyrum tataricum* starch during incubation. As shown in Fig. S4, quercetin significantly affected both the average pore size (*P* < 0.01) and specific surface area (*P* < 0.05) of *Fagopyrum tataricum* starch. Specifically, the BJH adsorption average pore size and BJH desorption average pore size decreased from 20.23 and 20.56 nm to 11.79 and 12.46 nm, respectively. Meanwhile, the BJH adsorption specific surface area and BJH desorption specific surface area decreased from 0.495 and 0.498 m^2^/g to 0.342 and 0.364 m^2^/g, respectively.

Additionally, Fig. 1c and d reveal the presence of substantial “flocculent debris” adhering to the surface of the starch granules. These fragments are formed by the cross-linking of quercetin molecules that did not penetrate the granules during incubation. However, this does not imply that these non-complexed quercetin molecules are “wasted” or fail to contribute to the increase in RS content. Research indicates that quercetin itself exerts an inhibitory effect on amylase enzymes (Qin et al., 2025; Shen et al., 2023). Thus, it is plausible that quercetin enhances the RS content of *Fagopyrum tataricum* through three concurrent mechanisms: (1) blocking pore structures, (2) complexing with starch molecules to create steric hindrance, and (3) inhibiting amylase activity. These mechanisms will be explored in greater detail in subsequent sections.

### 3.3 High-resolution 3D CT

In this study, high-resolution 3D CT technology was employed to observe starch structure, enabling layer-by-layer scanning of entire starch granules to visualize their internal structural characteristics. As shown in Fig. S5, the CT scans suggested that the outer region of *Fagopyrum tataricum* starch granules exhibited higher density, while the density progressively decreased toward the interior. Notably, the central part of some granules appeared completely hollow, displaying no detectable brightness signal. This observation suggests the presence of a distinct hollow internal structure within starch granules. In a separate study aimed at validation, 3D CT scanning was also performed on corn starch, one of the most common starches (Li et al., 2026). As presented in Fig. S6, corn starch granules, similar to *Fagopyrum tataricum* starch, exhibited dark internal regions, indicating extremely low density (or hollowness) within the granules of both starch types. Furthermore, potato starch, which possesses larger granules and a B-type crystalline structure, was selected for 3D CT analysis. As illustrated in Fig. S7, the results for potato starch differed markedly from those for *Fagopyrum tataricum* starch: a distinct bright band was observed on the granule surface, and while the internal density was lower, it was uniformly distributed. This contrasts sharply with the hollow (or extremely low-density) interior characteristic of corn and *Fagopyrum tataricum* starches. These findings suggest that some starch granules exhibit a hollow internal structure, whereas others are solid (Li et al., 2026). However, which starches are solid, which are hollow, and the underlying mechanisms governing this structural variation remain unknown, warranting further investigation.

**Fig. 2:**
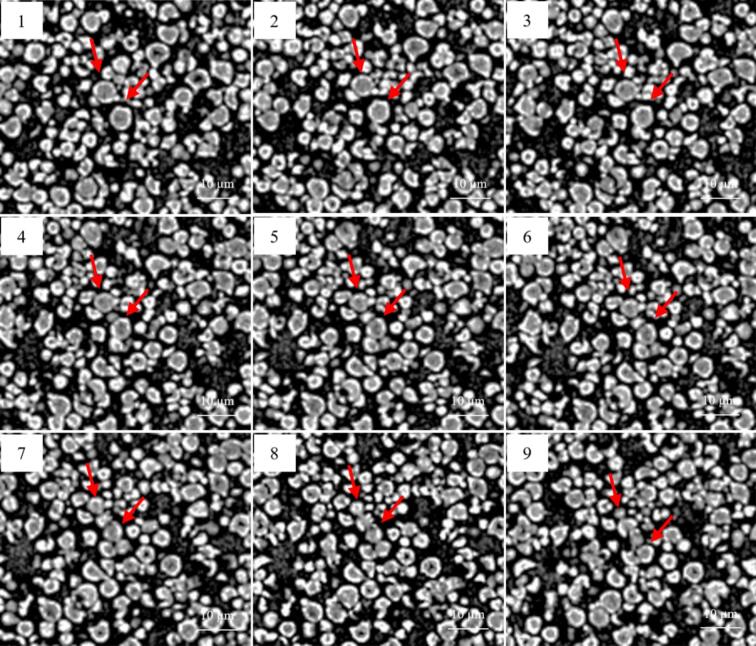
3D CT scanning of *Fagopyrum tataricum* starch-quercetin.

As depicted in Fig. 2, the vast majority of *Fagopyrum tataricum* starch granules incubated with quercetin exhibited a solid internal structure. For instance, the two granules indicated by red arrows, when examined via layer-by-layer CT scanning, did not display the hollow structure observed in Fig. S5. Nevertheless, a few granules in Fig. 2 still exhibited hollow characteristics, which may be attributable to incomplete incubation.

In summary, 3D CT observations suggested that native *Fagopyrum tataricum* starch granules possess a unique hollow internal structure. Following incubation with quercetin, a substantial amount of quercetin penetrated deep into the granule interior. The specific interactions occurring between the quercetin molecules that entered the granules and the starch molecules will be explored in greater detail in subsequent sections.

### 3.4 XRD

As shown in Fig. S8, XRD was employed to analyze the changes in the crystalline structure of *Fagopyrum tataricum* starch before and after quercetin incubation. The relative crystallinity of different starches typically ranges from 10% to 45%. Based on X-ray diffraction patterns, starches are generally classified into four crystalline types: A-type, B-type, C-type, and *V*-type (Bertoft et al., 2024). A-type starch exhibits characteristic diffraction peaks at 15°, 17°, 18°, and 23° (2θ), as observed in starches from wheat, corn, and rice. B-type starch displays characteristic peaks at approximately 5.6°, 17°, 22°, and 24° (2θ), such as those found in potato and cassava starches. C-type crystallinity represents a mixture of A- and B-type patterns, while V-type starch is characterized by distinct diffraction peaks resulting from the interaction of starch molecules with lipids, iodine, or other small molecules (Cardoso & Westfahl, 2010). The fundamental basis for starch crystallinity is the regular arrangement of double helices (Bertoft, 2017).

As presented in Fig. S8, native *Fagopyrum tataricum* starch exhibited a typical A-type crystalline pattern, with a relative crystallinity of 28.40%. Following incubation with quercetin, marked alterations in the crystalline structure were observed. Specifically, the original diffraction peaks at 15° and 23° disappeared, indicating that quercetin molecules penetrating the granules may interact with starch molecules, thereby modifying the original molecular arrangement.

### 3.5 FTIR

**Fig. 3:**
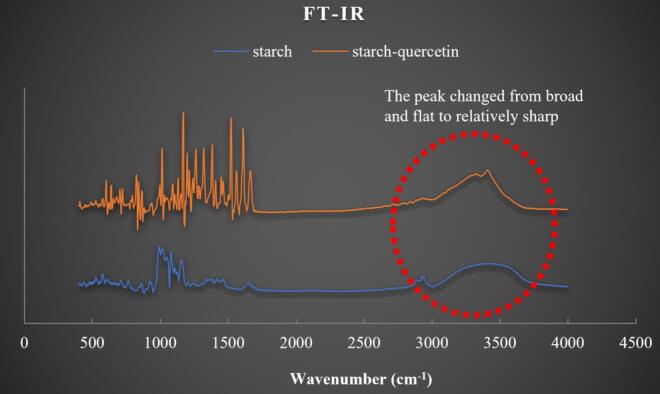
FT-IR results of *Fagopyrum tataricum* starch-quercetin complex.

The changes in the FTIR spectra of *Fagopyrum tataricum* starch before and after quercetin incubation were analyzed. In the FTIR spectrum, the region between 800 and 1200 cm^−1^ corresponds to the fingerprint region of starch (Gao et al., 2021; Teixeira et al., 2018). As shown in Fig. 3, after incubation with quercetin, the fingerprint region of *Fagopyrum tataricum* starch exhibited substantial changes, which are closely associated with the introduction of quercetin. The broad absorption band between 3000 and 3600 cm^−1^ is attributed to O—H stretching vibrations, which can reflect the strength and number of hydrogen bonds within the starch molecules (Bokobza, 2019). Hydroxyl groups with stronger hydrogen bonding exhibit lower vibrational frequencies, with absorption occurring at lower wavenumbers (approximately 3200 cm^−1^), whereas those with weaker hydrogen bonding display higher vibrational frequencies, with absorption shifting to higher wavenumbers (approximately 3400 cm^−1^). (Bokobza, 2019).

As shown in Fig. 3, the shape of the O—H stretching band (3000–3600 cm^−1^) changed markedly after quercetin incubation, indicating the formation of new hydrogen bonds between quercetin and starch molecules and a possible reorganization of the hydrogen bonding network in the system. Notably, in native starch, only starch-starch and starch-water hydrogen bonds exist. After quercetin incorporation, multiple types of hydrogen bonds may be present, including starch-quercetin, quercetin-water, starch-starch, starch-water, and quercetin-quercetin interactions. However, as shown in Fig. 3, the O—H stretching band shifted from a broad, flat profile to a sharper peak centered at approximately 3400 cm^−1^ after quercetin incubation. This suggests that the introduction of quercetin may lead to a more homogeneous hydrogen bonding network throughout the system.

### 3.6 DSC

DSC was employed to analyze the changes in gelatinization temperature and crystalline melting enthalpy (ΔH) of *Fagopyrum tataricum* starch before and after quercetin incubation. As shown in Fig. S8, native *Fagopyrum tataricum* starch exhibited a ΔH of 12.33 J/g, with an onset temperature (To) of 64.3 °C, a peak temperature (Tp) of 69.2 °C, and a conclusion temperature (Tc) of 75.3 °C. Following quercetin incubation, the ΔH of *Fagopyrum tataricum* starch decreased to 6.893 J/g, and To, Tp, and Tc were reduced to 62.9 °C, 67.6 °C, and 73.2 °C, respectively. Thus, quercetin incubation resulted in a decrease in both the gelatinization temperature and the crystalline melting enthalpy of *Fagopyrum tataricum* starch. It should be noted that the crystalline melting temperature of quercetin is approximately 313 °C (Wang et al., 2022, Wang, Liu, Liu, Guo and Li, 2022). Since the present study focused primarily on starch, the scanning temperature range was set from 40 to 97 °C; consequently, no melting peak corresponding to quercetin crystals was observed in Fig. S8. Admittedly, in this study, only a 5% concentration of quercetin was used for characterizations such as SEM, XRD, and DSC. Whether the effects of quercetin on the structure and properties of *Fagopyrum tataricum* starch at other concentrations fully follow the same trend remains to be further investigated.

In conclusion, structure dictates properties. The incorporation of quercetin may alter the hydrogen bonding network and its strength within native *Fagopyrum tataricum* starch, leading to significant changes in its physicochemical properties. The specific molecular interactions between quercetin and starch molecules will be further explored in the following section.

### 3.7 Interaction between starch-quercetin complex and α-amylase

(1) Interaction between quercetin and starch

The aforementioned XRD, DSC, FTIR, and SEM results suggested that quercetin molecules could penetrate into *Fagopyrum tataricum* starch granules during incubation, interact with starch molecules, and subsequently alter the digestion and gelatinization properties of the starch. However, the detailed molecular interactions between quercetin and starch remained unclear. In this section, molecular docking and simulation calculations were employed to elucidate the interaction details between quercetin and starch, as well as the underlying mechanisms by which quercetin enhances the RS content of *Fagopyrum tataricum*.

**Fig. 4:**
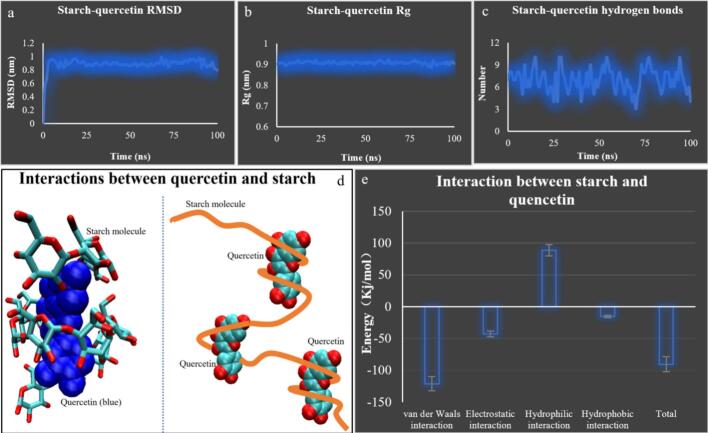
Interactions between starch and quercetin.

As illustrated in Fig. 4, when starch molecules approached quercetin, the starch chains gradually coiled around the quercetin molecule (up to two turns), in a manner analogous to starch–lipid interactions (Chen et al., 2020; Cheng et al., 2018). The most notable difference is that no hydrogen bonds are formed during starch–lipid interactions. In contrast, due to the presence of six exposed hydroxyl groups on the quercetin molecule, numerous intermolecular hydrogen bonds were formed between quercetin and starch. As shown in Fig. 4a and b, the relatively small fluctuations in Rg and RMSD values during the simulation indirectly suggest that the binding between quercetin and starch is relatively stable. As shown in Fig. 4c, during the 100 ns simulation, the number of intermolecular hydrogen bonds remained consistently around seven (each hydroxyl group can act simultaneously as both a hydrogen bond donor and acceptor). In addition to hydrogen bonding, secondary interactions of approximately 90 kJ/mol were observed between quercetin and the starch molecule (comprising 12 glucose residues), compared to the bond energy of a carbon–carbon single bond (approximately 330 kJ/mol). These results indicate that the interaction between quercetin and starch is relatively stable, and the complex can form spontaneously in aqueous solution (the negative total binding free energy in Fig. 4e confirms spontaneous complexation).

(2) Structural characteristics of α-amylase

Prior to introducing the interaction between the starch-quercetin complex and α-amylase, the tertiary structural features of α-amylase are briefly described to facilitate reader understanding. As shown in Fig. S9, human α-amylase is composed of 14 α-helices and 8 β-sheets, with a radius of gyration (Rg) of 2.33 nm, a solvent-accessible surface area of 193.7 nm^2^, a molecular weight of 55 kDa, and an isoelectric point (pI) of 6.87 (Zhong, She, et al., 2024b).

As illustrated in Fig. S9, the surface of α-amylase features an active cleft approximately 3.5 nm in length and 1.5 nm in width, which is responsible for binding starch chains. Located in the central region of this active cleft is an active groove with a depth of approximately 1.4 nm, at the bottom of which resides the catalytic triad (Asp197-Glu233-Asp300) (Zhong, She, et al., 2024b). Within this triad, Glu233 initiates catalysis by releasing a proton to attack the glycosidic oxygen atom; Asp197 is involved in the formation and stabilization of the oxocarbenium ion intermediate; and Asp300 assists Glu233 in extracting a proton from a water molecule to generate an activated hydroxyl group by stabilizing the nucleophilic water molecule, ultimately enabling the hydroxyl group to attack the oxocarbenium ion intermediate and complete hydrolysis (Mandal et al., 2019). Additionally, six highly flexible loops surrounding the active cleft may be closely associated with substrate recognition and binding.

(3) Interaction between starch-quercetin complex and α-amylase

**Fig. 5:**
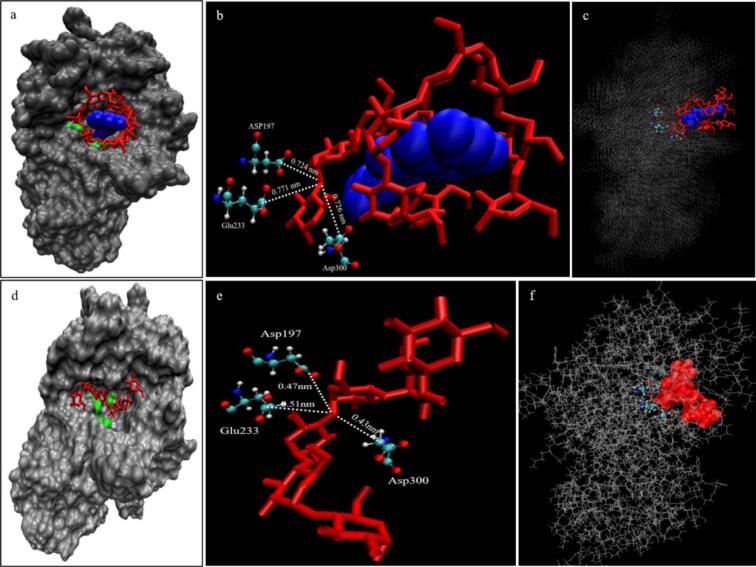
Molecular docking between α-amylase and starch-quercetin complex (d, e, f: Control group for starch-α-amylase interaction).

To elucidate the mechanism underlying the increased RS content in *Fagopyrum tataricum* following quercetin introduction, molecular docking and simulations were employed to further analyze the interaction details between the starch–quercetin complex and α-amylase. As shown in Fig. 5, when a single starch chain interacted with α-amylase, the entire starch chain adopted a “flat” conformation within the active cleft of the enzyme (Fig. 5d). Specifically, the third and fourth glucose residues penetrated deeply into the active groove. Following docking, the distances between the glycosidic oxygen atom located between G3 and G4 and the carboxyl groups of Asp197, Glu233, and Asp300 were 0.47 nm, 0.51 nm, and 0.43 nm, respectively (Fig. 5e). As illustrated in Fig. 5a, due to steric hindrance, the starch chain complexed with quercetin could not fully insert into the active groove of α-amylase, thereby failing to establish adequate contact with the catalytic triad. The distances between the glycosidic oxygen atom nearest to the active center and the carboxyl groups of Asp197, Glu233, and Asp300 were 0.724 nm, 0.771 nm, and 0.726 nm, respectively (Fig. 5b)-distances insufficient to support nucleophilic attack on the glycosidic bond by the catalytic triad of α-amylase (Goldbach et al., 2019; Zhong, She, et al., 2024c). This may be the second reason why the introduction of quercetin increases the RS content of *Fagopyrum tataricum*.

Furthermore, the interaction sites between the starch-quercetin complex and α-amylase were primarily localized to flexible loops 3 and 5 of the enzyme, whereas the terminal residues of the free starch chain primarily interacted with flexible loops 1 and 4 (Fig. 5 and Fig. S9c).

As shown in Fig. S10 and S11, molecular dynamics simulations were employed to analyze the stability of the interaction between the starch–quercetin complex and α-amylase. Throughout the 100 ns simulation, the starch–quercetin complex remained consistently bound within the active groove of α-amylase, without dissociation or significant conformational changes (Fig. S10c). This further demonstrates the high stability of both the starch–quercetin interaction and the complex–enzyme interaction. The distances between the glycosidic oxygen atom nearest to the active center and the carboxyl groups of Asp197, Glu233, and Asp300 remained relatively constant at approximately 0.73 nm, 0.78 nm, and 0.70 nm, respectively, indicating that the quercetin-complexed starch chain was not correctly recognized by α-amylase throughout the simulation (Fig. S10b).

The interaction energies between the starch-quercetin complex and α-amylase were calculated using the MM-PBSA method. The results indicated that the total interaction energy between the free starch chain and α-amylase was approximately 78 kJ/mol, whereas that between the starch-quercetin complex and α-amylase was approximately 53 kJ/mol (Fig. S11a). Among these interactions, van der Waals forces accounted for approximately 68.7%, and electrostatic interactions contributed approximately 22.5%. Notably, despite the larger molecular weight of the starch–quercetin complex compared to the free starch chain (the models used in this study), its interaction energy with α-amylase was lower. This may be attributed to the pre-existing interaction between starch and quercetin, as well as the less extensive contact area between the complex and α-amylase relative to the free starch chain. Additionally, residues such as Trp59, Tyr151, Thr163, and Ala307 in α-amylase played important roles in stabilizing the interaction with the starch-quercetin complex (Fig. S11b).

Hydrogen bonding also constitutes a significant type of secondary interaction, with each hydrogen bond contributing approximately 35 kJ/mol of bond energy (Jabłoński, 2023). Both quercetin and starch molecules possess numerous hydroxyl groups, and the vicinity of the α-amylase active groove contains multiple amino acid residues with R groups capable of forming hydrogen bonds (e.g., -OH or -COOH), including Asn53, Gln63, Ile148, Thr151, and Thr163 (Fig. S11c). During binding with the quercetin–starch complex, Thr163, Gln63, and Trp59 exhibited the highest frequency of hydrogen bond formation (Fig. S11d), indicating their crucial roles in complex stabilization.

For quercetin, the starch matrix can protect it from degradation by gastric acid, thereby enabling sustained release in the small intestine and targeted delivery to the colon. In the small intestine, a portion of quercetin can be directly absorbed and utilized by intestinal epithelial cells. In the colon, quercetin can be metabolized by the gut microbiota into active small molecules, such as DOPAC, which enhance antioxidant, anti-inflammatory, and immunomodulatory effects (Li, Zhang, & Wang, 2025).

In summary, starch molecules and quercetin spontaneously form stable complexes in aqueous solution, with a binding free energy of approximately 90 kJ/mol and the involvement of approximately seven hydrogen bonds. Due to steric hindrance, the quercetin-complexed starch chain cannot fully insert into the active groove of α-amylase to adequately contact the catalytic triad: a key factor underlying the altered digestion rate of *Fagopyrum tataricum* starch upon quercetin introduction. Furthermore, this starch–quercetin complex not only evades correct recognition by α-amylase but also occupies the enzyme's active groove, engaging in an interaction of approximately 53 kJ/mol with α-amylase. This occupation further inhibits the hydrolysis of other starch molecules by α-amylase, thereby contributing to the increased RS content.

### 3.8 Interaction between quercetin and α-amylase

As observed in Fig. 1c, substantial flocculent debris was present on the surface of starch granules, formed by the cross-linking of quercetin molecules that did not penetrate into the granules. As previously noted, although these quercetin molecules did not enter the starch granules, they may still contribute to the increase in RS content. Experimental evidence has demonstrated that quercetin exerts an inhibitory effect on α-amylase (Qin et al., 2025; Shen et al., 2023). In this study, molecular docking and simulations were employed to analyze the interaction details between quercetin and α-amylase.

**Fig. 6:**
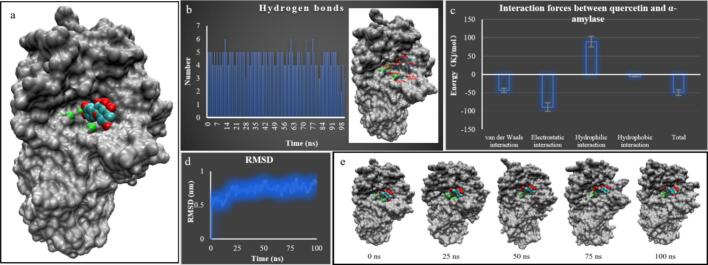
Interaction between quercetin and α-amylase (a: molecular docking; b: hydrogen bonding; c: interaction forces; d: RMSD during simulation; e: conformational changes during simulation).

As shown in Fig. 6, quercetin molecules can be misrecognized by α-amylase and bind within its active groove, thereby hindering the binding of starch molecules and exerting an inhibitory effect on α-amylase (Fig. 6a). Upon binding within the active groove of α-amylase, quercetin formed hydrogen bonds with surrounding residues, including Trp59, Gln62, Gln63, Arg303, and Asp356, as well as with two residues of the catalytic triad, Asp197 and Asp300 (Fig. 6b). Calculations indicated that the number of hydrogen bonds between quercetin and α-amylase remained consistently around five throughout the 100 ns simulation (Fig. 6b). In addition to hydrogen bonding, secondary interactions of approximately 49.4 kJ/mol were observed between quercetin and α-amylase (Fig. 6c), comprising van der Waals forces (approximately 31%) and electrostatic interactions (approximately 64%). These results indicate that the interaction between quercetin and α-amylase is highly stable. As illustrated in Fig. 6e, quercetin molecules did not dissociate from the active groove of α-amylase throughout the entire 100 ns simulation.

In summary, quercetin molecules can stably bind within the active groove of α-amylase through strong hydrogen bonding, van der Waals forces, and electrostatic interactions, thereby obstructing the binding of starch molecules to the enzyme. This may be the third reason why the introduction of quercetin increases the RS content of *Fagopyrum tataricum*.

Nevertheless, the present molecular simulation results were obtained under idealized in silico conditions, which may not fully reflect the complex microenvironment, dynamic conformational changes, and multifactorial interactions occurring in actual physiological or food systems.

## 4 Conclusion

In this study, the interaction between quercetin and *Fagopyrum tataricum* starch, as well as the mechanism by which it increases the RS content, was analyzed. The main findings are summarized as follows: First, the incorporation of quercetin significantly increased the RS content of *Fagopyrum tataricum*. At a quercetin addition level of 5%, the RS content was elevated to 60.38%.

Second, SEM and 3D CT characterization suggested that quercetin penetrated into the starch granules and blocked the pore structures within *Fagopyrum tataricum* starch. This blockage reduced the specific surface area of the starch granules, making it more difficult for amylase to penetrate the granules for hydrolysis. This may be the first reason why quercetin increases RS content.

Third, DSC, XRD, and FTIR analyses suggested that quercetin penetrating the starch granules interacted with starch molecules, forming new hydrogen bonds and reconstructing the original hydrogen bonding network. This resulted in a more homogeneous hydrogen bonding system within the starch-quercetin complex (evidenced by the O—H stretching band at 3000–3600 cm^−1^ shifting from a broad, flat profile to a sharper peak) and altered the molecular arrangement of starch (as indicated by the disappearance of the original diffraction peaks at 15° and 23°). Concurrently, both the crystalline melting enthalpy and gelatinization temperature of *Fagopyrum tataricum* starch were reduced.

Fourth, molecular simulations indicated that starch molecules and quercetin spontaneously formed stable complexes in aqueous solution, with a binding free energy of approximately 90 kJ/mol and the involvement of approximately 7 hydrogen bonds. Due to steric hindrance, the quercetin-complexed starch chain could not fully insert into the active groove of α-amylase to adequately contact the catalytic triad. This may be the second reason why quercetin increases RS content.

Fifth, quercetin molecules that did not penetrate the starch granules could stably bind within the active groove of α-amylase through strong hydrogen bonding, van der Waals forces, and electrostatic interactions, thereby obstructing the binding of starch molecules to the enzyme and exerting an inhibitory effect on α-amylase. This may be the third reason why quercetin increases RS content.

However, the specific contribution of each mechanism to RS remains to be further investigated. In addition to the three mechanisms described above, quercetin, as a small molecule rich in hydroxyl groups, may also act as a cross-linking agent following starch gelatinization and granule disintegration. By forming hydrogen bonds with starch molecules, it could serve as a bridge between starch chains, thereby accelerating molecular rearrangement in solution and promoting starch retrogradation. This proposed mechanism warrants further investigation.

The results will provide important references for the preparation of RS5 and lay a crucial foundation for the recycling of discarded *Fagopyrum tataricum* hull resources.

## CRediT authorship contribution statement

**Shengchun Li:** Writing – review & editing, Writing – original draft, Data curation. **Haixia Zhong:** Writing – review & editing, Writing – original draft, Data curation. **Anhu Wang:** Supervision, Funding acquisition, Formal analysis. **Qiuping Wei:** Data curation. **Lan Wang:** Data curation. **Wenhui Ma:** Writing – review & editing, Writing – original draft, Supervision. **Zhiguang Chen:** Writing – review & editing, Writing – original draft, Supervision.

## Declaration of competing interest

The authors declare that they have no known competing financial interests or personal relationships that could have appeared to influence the work reported in this paper.

## Data Availability

The data that has been used is confidential.
