# Supplementary material for: Mechanistic study of quercetin on Fagopyrum Tataricum resistant starch
Source: Food Chem X. 2026 Apr 20;35:103883. doi: 10.1016/j.fochx.2026.103883 (PMC13126029; doi:10.1016/j.fochx.2026.103883)
Supplement: Supplementary file 1 — Supplementary material 1 [file mmc1.pdf]

ASAP 2460 Version 3.01

Sample: CZG#1
Operator: jch
Submitter: s/n 102
Bar Code:
File: E:\ZZJJ\20260415

Started: 2026/4/13 07:59:35
Completed: 2026/4/13 11:05:15
Report time: 2026/4/13 11:07:04
Sample mass: 1.4588 g
Analysis free space: 40.9780 cm^3
Low pressure dose: None
Automatic degas: No

Analysis adsorptive: N_2
Analysis bath temp: -195.800 ℃
Thermal correction: No
Ambient free space: 14.8086 cm^3 Measured
Equilibration interval: 30 s
Sample density: 1.000 g/cm^3

Pore size

Adsorption average pore diameter (4V/A by BET): 10.3762 nm
Desorption average pore diameter (4V/A by BET): 1.1408 nm
BJH Adsorption average pore width (4V/A): 11.1664 nm
BJH Desorption average pore width (4V/A): 12.6192 nm

Surface Area

BET Surface Area: 0.4504 m^2/g
BJH Adsorption cumulative surface area: 0.3057 m^2/g
BJH Desorption cumulative surface area: 0.3530 m^2/g

Horvath-Kawazoe

Maximum pore volume at $p/p°$ = 0.000000000: 0.000000 cm^3/g
Median pore width: 0.0000 nm

ASAP 2460 Version 3.01

Sample: CZG#2

Operator: jch

Submitter: s/n 102

Bar Code:

File: E:\ZZJJ\20260415

Started: 2026/4/13 11:39:26

Completed: 2026/4/13 14:45:04

Report time: 2026/4/13 14:48:11

Sample mass: 1.3696 g

Analysis free space: 42.1315 cm^3

Low pressure dose: None

Automatic degas: No

Analysis adsorptive: N2

Analysis bath temp: -195.800 ℃

Thermal correction: No

Ambient free space: 15.0132 cm^3 Measured

Equilibration interval: 30 s

Sample density: 1.000 g/cm^3

Pore size

Adsorption average pore diameter (4V/A by BET): 10.1754 nm

Desorption average pore diameter (4V/A by BET): 1.1003 nm

BJH Adsorption average pore width (4V/A): 11.3544 nm

BJH Desorption average pore width (4V/A): 12.0162 nm

Surface Area

BET Surface Area: 0.4372 m^2/g

BJH Adsorption cumulative surface area: 0.3189 m^2/g

BJH Desorption cumulative surface area: 0.3245 m^2/g

Horvath-Kawazoe

Maximum pore volume at $p/p° = 0.000000000$: 0.000000 cm^3/g

Median pore width: 0.0000 nm

ASAP 2460 Version 3.01

Sample: CZG#3

Operator: jch

Submitter: s/n 102

Bar Code:

File: E:\ZZJJ\20260415

Started: 2026/4/13 15:04:13

Completed: 2026/4/13 18:12:10

Report time: 2026/4/13 18:15:23

Sample mass: 1.5546 g

Analysis free space: 44.0382 cm^3

Low pressure dose: None

Automatic degas: No

Analysis adsorptive: N_2

Analysis bath temp: -195.800 ℃

Thermal correction: No

Ambient free space: 15.1804 cm^3 Measured

Equilibration interval: 30 s

Sample density: 1.000 g/cm^3

Pore size

Adsorption average pore diameter (4V/A by BET): 11.5255 nm

Desorption average pore diameter (4V/A by BET): 1.2538 nm

BJH Adsorption average pore width (4V/A): 12.8594 nm

BJH Desorption average pore width (4V/A): 12.7467 nm

Surface Area

BET Surface Area: 0.4951 m^2/g

BJH Adsorption cumulative surface area: 0.4039 m^2/g

BJH Desorption cumulative surface area: 0.4166 m^2/g

Horvath-Kawazoe

Maximum pore volume at $p/p°$ = 0.000000000: 0.000000 cm^3/g

Median pore width: 0.0000 nm

ASAP 2460 Version 3.01

Sample: CZG#4
Operator: jch
Submitter: s/n 102
Bar Code:
File: E:\ZZJJ\20260416

Started: 2026/4/14 08:11:55
Completed: 2026/4/14 11:26:58
Report time: 2026/4/14 11:34:15
Sample mass: 1.5138 g
Analysis free space: 42.1466 cm^3
Low pressure dose: None
Automatic degas: No

Analysis adsorptive: N_2
Analysis bath temp: -195.800 ℃
Thermal correction: No
Ambient free space: 16.0152 cm^3 Measured
Equilibration interval: 30 s
Sample density: 1.000 g/cm^3

Pore size

Adsorption average pore diameter (4V/A by BET): 17.1338 nm
Desorption average pore diameter (4V/A by BET): 1.7802 nm
BJH Adsorption average pore width (4V/A): 19.9154 nm
BJH Desorption average pore width (4V/A): 20.1418 nm

Surface Area

BET Surface Area: 0.5376 m^2/g
BJH Adsorption cumulative surface area: 0.4732 m^2/g
BJH Desorption cumulative surface area: 0.4745 m^2/g

Horvath-Kawazoe

Maximum pore volume at $p/p° = 0.000000000$: 0.000000 cm^3/g
Median pore width: 0.0000 nm

ASAP 2460 Version 3.01

Sample: CZG#5

Operator: jch

Submitter: s/n 102

Bar Code:

File: E:\ZZJJ\20260416

Started: 2026/4/14 11:44:08

Completed: 2026/4/14 14:58:20

Report time: 2026/4/14 15:01:37

Sample mass: 1.5167 g

Analysis free space: 41.5477 cm³

Low pressure dose: None

Automatic degas: No

Analysis adsorptive: N2

Analysis bath temp: -195.800 ℃

Thermal correction: No

Ambient free space: 15.2528 cm³ Measured

Equilibration interval: 30 s

Sample density: 1.000 g/cm³

Pore size

Adsorption average pore diameter (4V/A by BET): 18.4781 nm

Desorption average pore diameter (4V/A by BET): 1.8130 nm

BJH Adsorption average pore width (4V/A): 21.0124 nm

BJH Desorption average pore width (4V/A): 21.4519 nm

Surface Area

BET Surface Area: 0.6018 m²/g

BJH Adsorption cumulative surface area: 0.5199 m²/g

BJH Desorption cumulative surface area: 0.5174 m²/g

Horvath-Kawazoe

Maximum pore volume at p/p° = 0.000000000: 0.000000 cm³/g

Median pore width: 0.0000 nm

ASAP 2460 Version 3.01

Sample: CZG#6
Operator: jch
Submitter: s/n 102
Bar Code:
File: E:\ZZJJ\20260416

Started: 2026/4/14 15:25:19
Completed: 2026/4/14 18:39:54
Report time: 2026/4/14 18:51:57
Sample mass: 1.4766 g
Analysis free space: 42.1398 cm^3
Low pressure dose: None
Automatic degas: No

Analysis adsorptive: N_2
Analysis bath temp: -195.800 ℃
Thermal correction: No
Ambient free space: 14.8514 cm^3 Measured
Equilibration interval: 30 s
Sample density: 1.000 g/cm^3

Pore size

Adsorption average pore diameter (4V/A by BET): 17.2771 nm
Desorption average pore diameter (4V/A by BET): 1.6853 nm
BJH Adsorption average pore width (4V/A): 19.7867 nm
BJH Desorption average pore width (4V/A): 20.1159 nm

Surface Area

BET Surface Area: 0.5612 m^2/g
BJH Adsorption cumulative surface area: 0.4910 m^2/g
BJH Desorption cumulative surface area: 0.5016 m^2/g

Horvath-Kawazoe

Maximum pore volume at $p/p°$ = 0.000000000: 0.000000 cm^3/g
Median pore width: 0.0000 nm
