# Supplementary material for: Mechanistic study of quercetin on Fagopyrum Tataricum resistant starch
Source: Food Chem X. 2026 Apr 20;35:103883. doi: 10.1016/j.fochx.2026.103883 (PMC13126029; doi:10.1016/j.fochx.2026.103883)
Supplement: Supplementary file 2 — Supplementary material 2 [file mmc2.docx]

**Supplementary material**

Fig. S1 Addition of quercetin to *Fagopyrum tataricum* flour to enhance RS content

Fig. S2 Initial models

Fig. S3 Effect of quercetin addition on the RS and hydrolysis rate of *Fagopyrum tataricum* starch

Fig. S4 Effect of quercetin on the pore size and specific surface area of *Fagopyrum tataricum* starch

Fig. S5 3D CT scanning of *Fagopyrum tataricum* starch

Fig. S6 3D CT scanning of corn starch

Fig. S7 3D CT scanning of potato starch

Fig. S8 XRD and DSC results of *Fagopyrum tataricum* starch-quercetin complex

Fig. S9 Interaction sites between starch-quercetin complex and α-amylase

Fig. S10 Atomic distances, RMSD, and conformational changes of starch-quercetin-α-amylase complex during molecular simulation

Fig. S11 Interaction forces between starch-quercetin complex and α-amylase


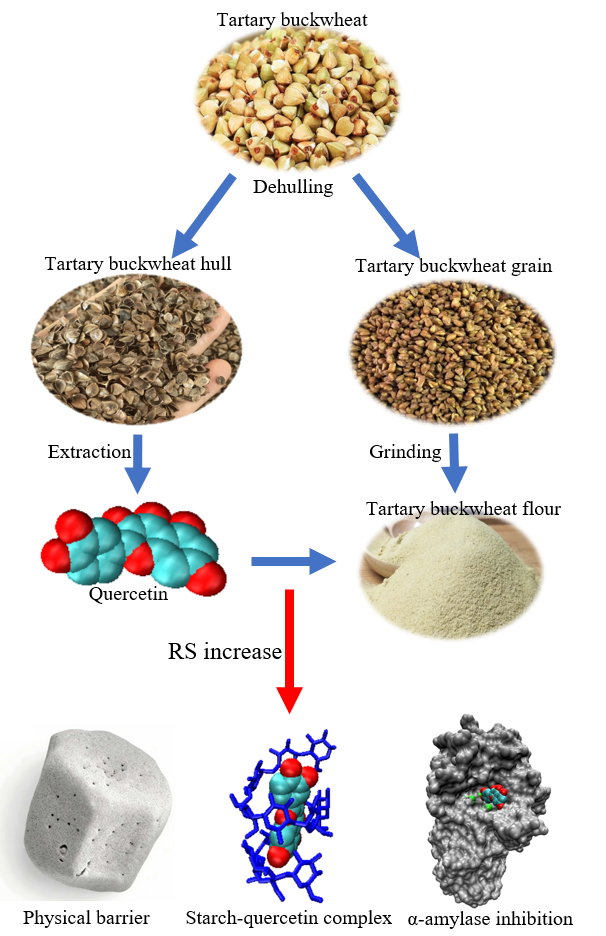


Fig. S1 Addition of quercetin to *Fagopyrum tataricum* flour to enhance RS content


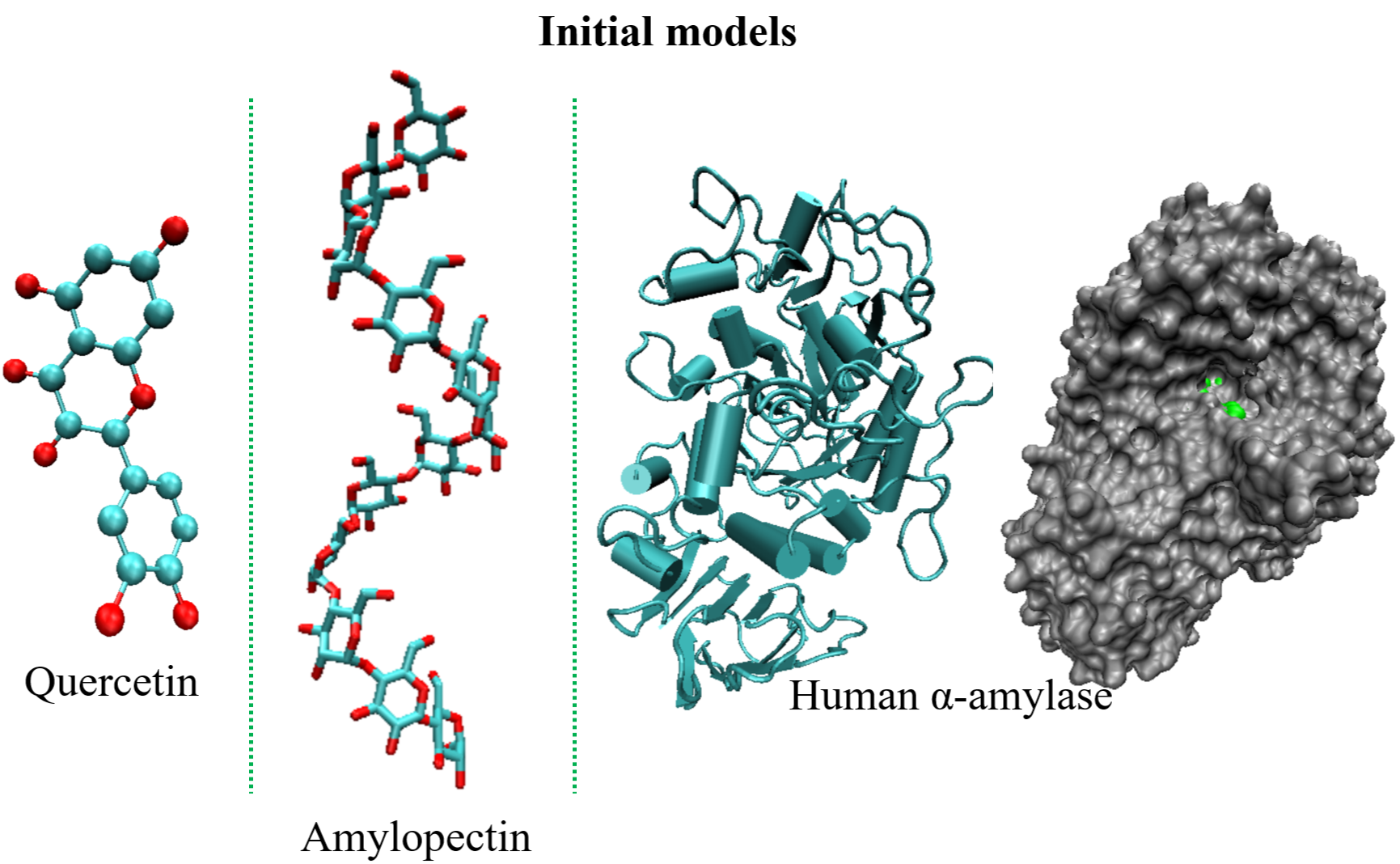


Fig. S2 Initial models


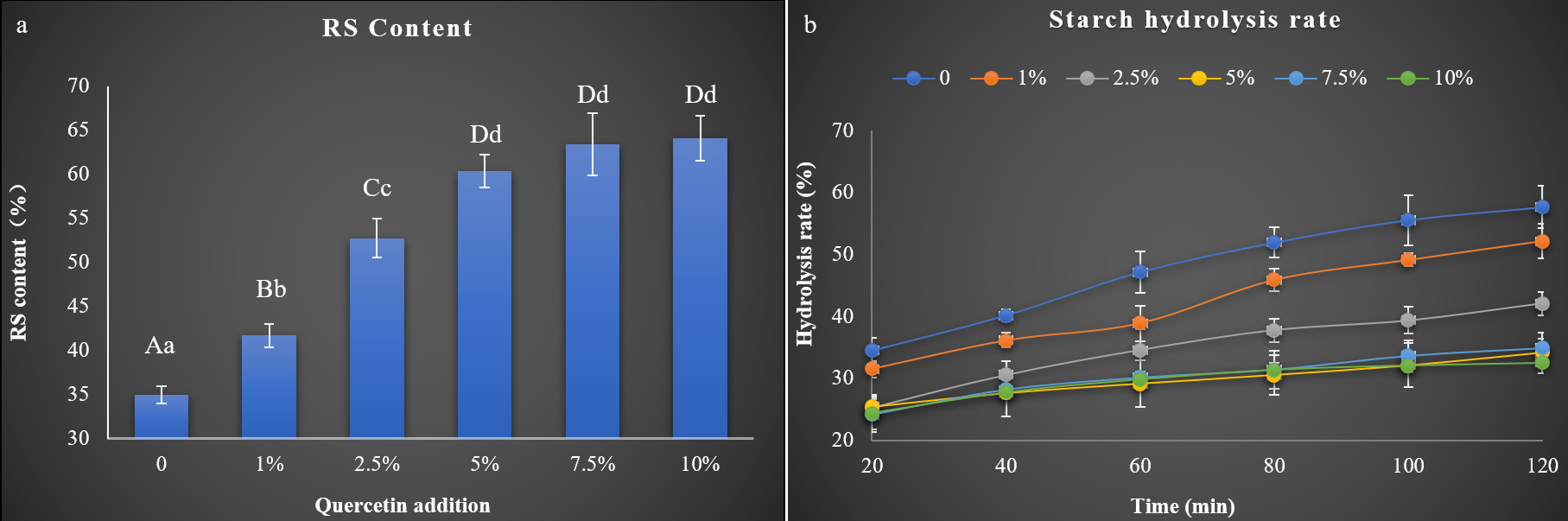


Fig. S3 Effect of quercetin addition on the RS and hydrolysis rate of *Fagopyrum tataricum*

(Different uppercase letters indicate P<0.01; different lowercase letters indicate P<0.05)


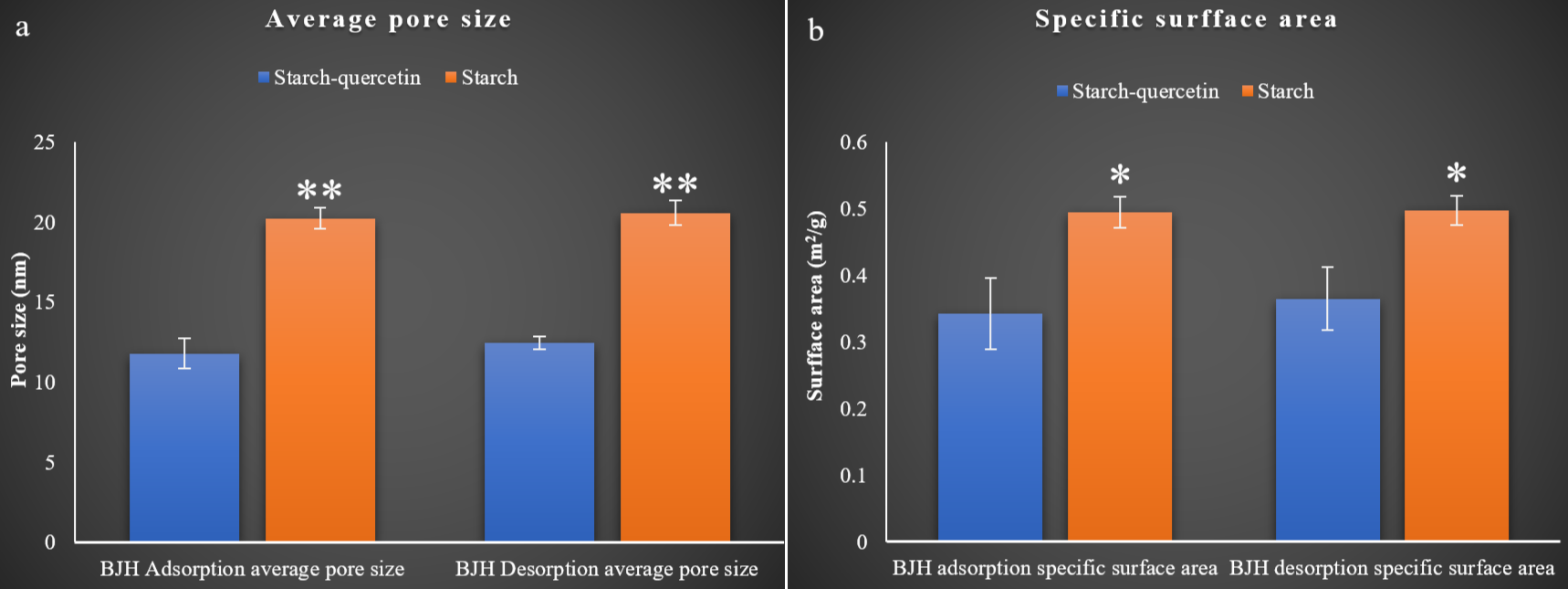


Fig. S4 Effect of quercetin on the pore size and specific surface area of *Fagopyrum tataricum* starch

(**: P<0.01; *: P<0.05)


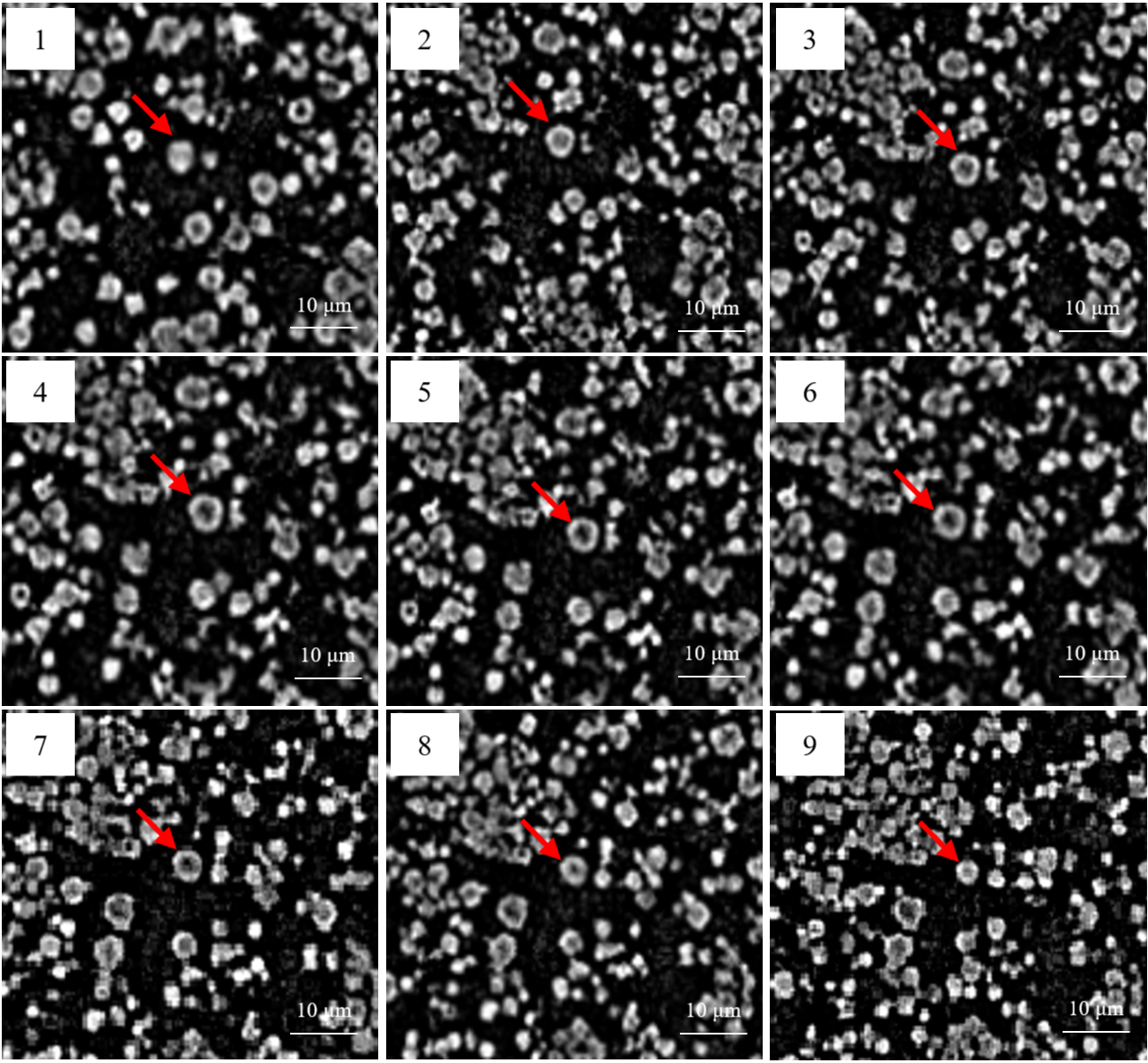


Fig. S5 3D CT scanning of *Fagopyrum tataricum* starch


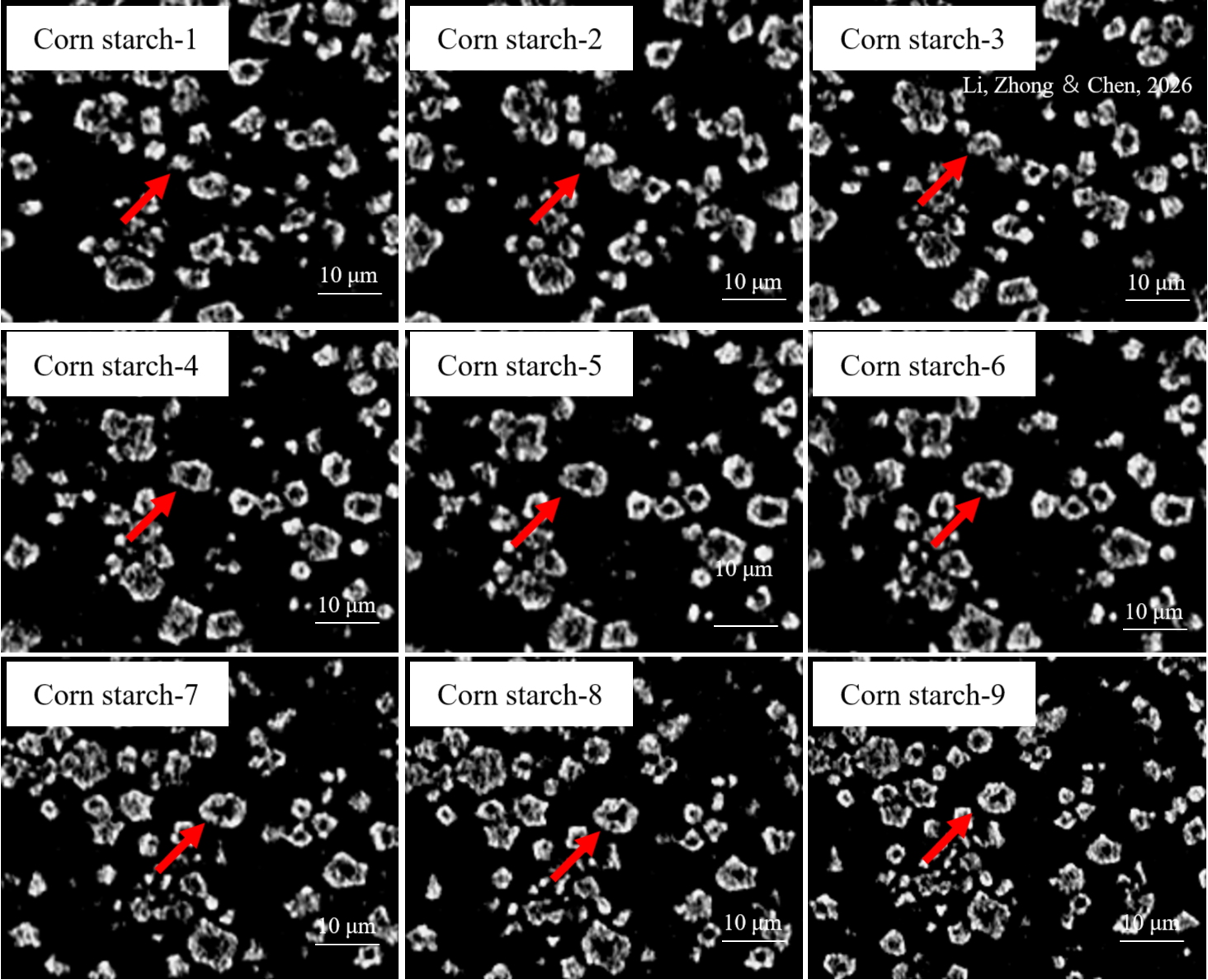


Fig. S6 3D CT scanning of corn starch


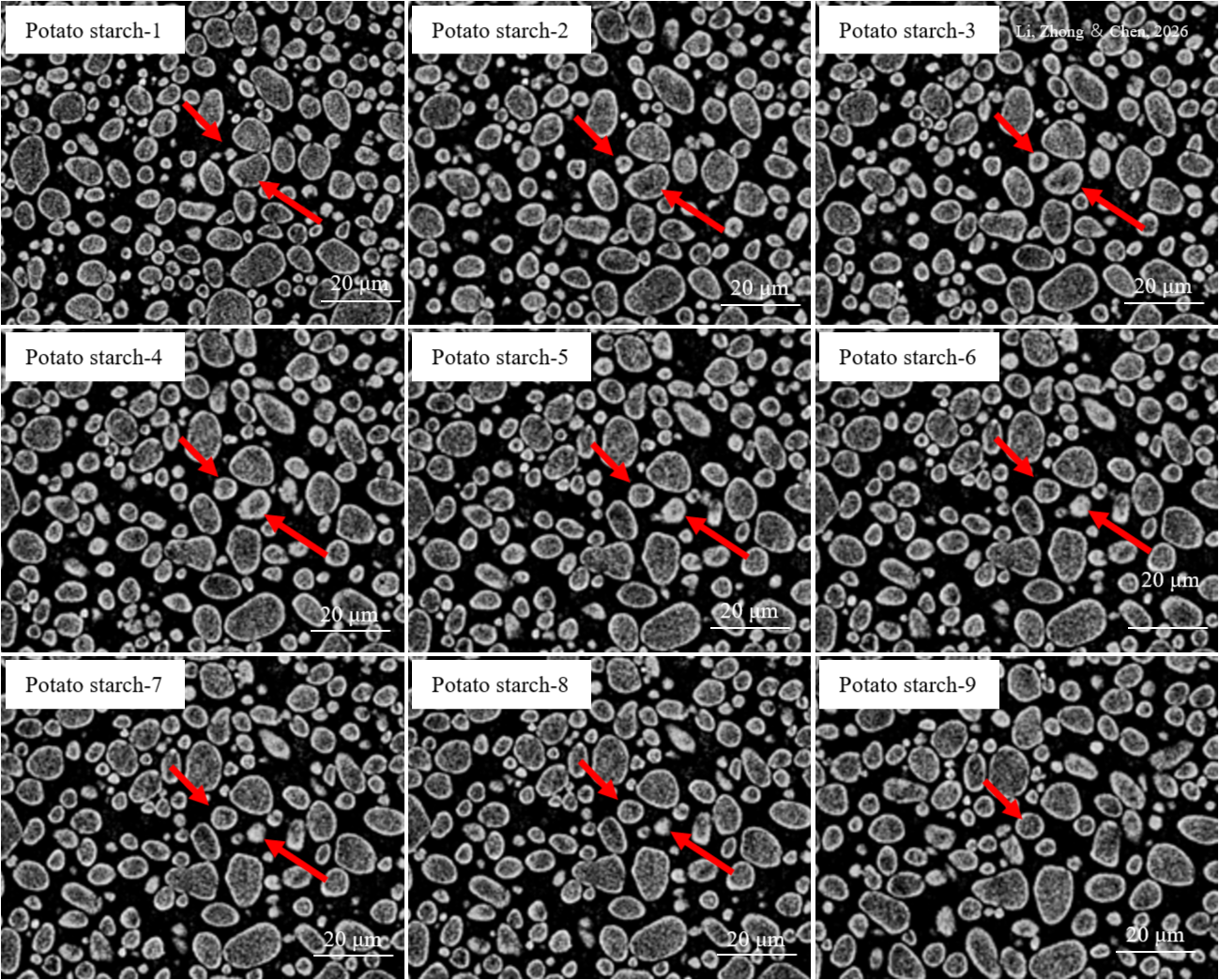


Fig. S7 3D CT scanning of potato starch


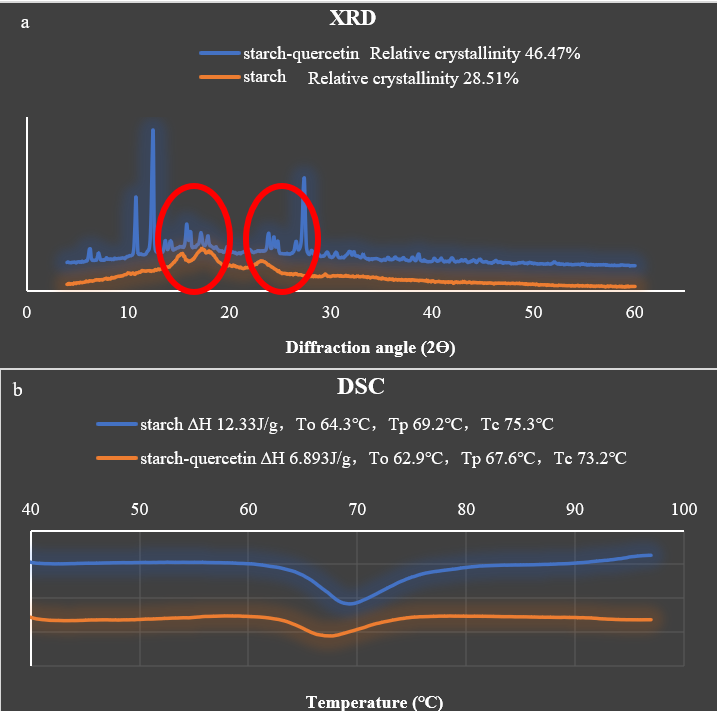


Fig. S8 XRD and DSC results of *Fagopyrum tataricum* starch-quercetin complex


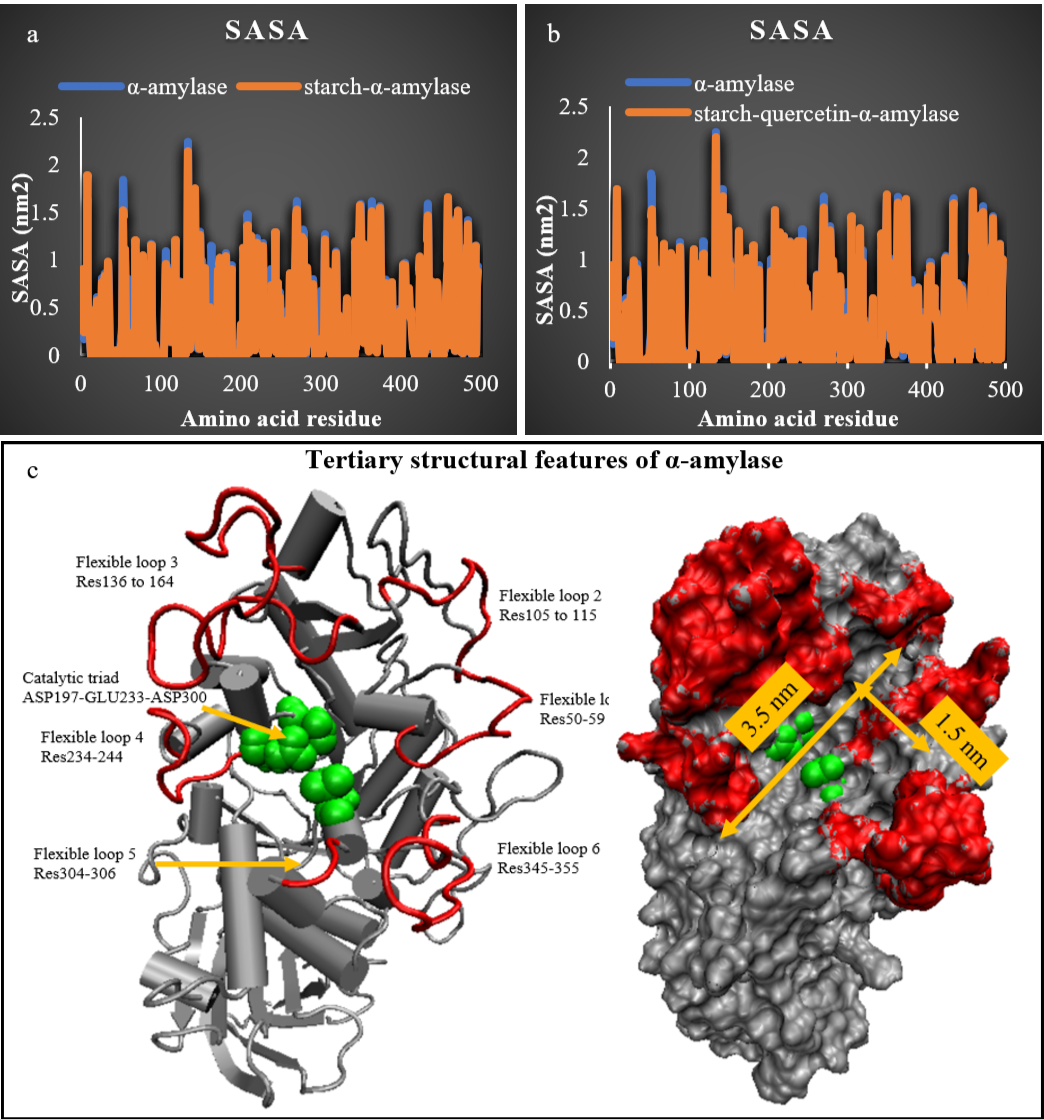


Fig. S9 Interaction sites between starch-quercetin complex and α-amylase


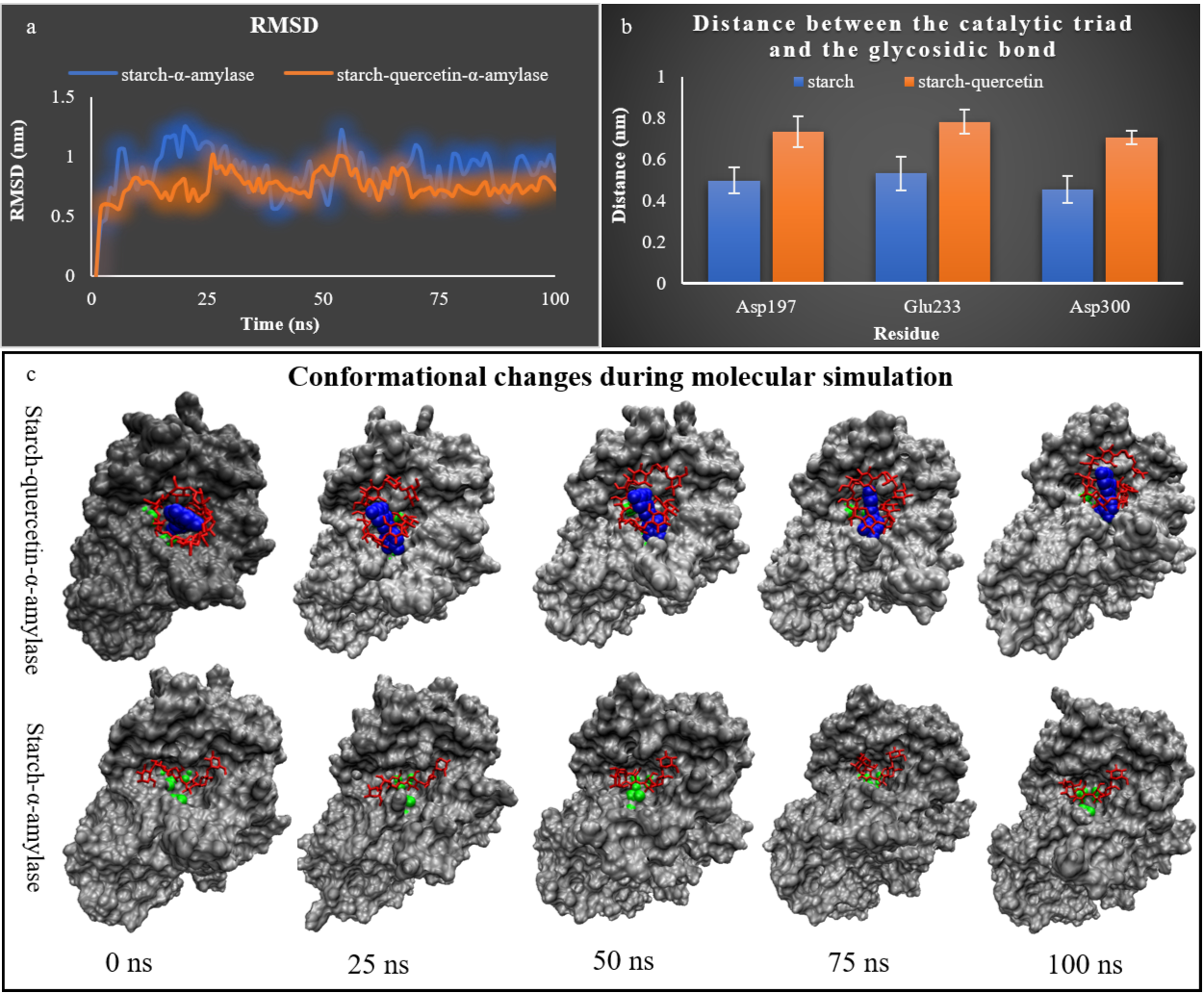


Fig. S10 Atomic distances, RMSD, and conformational changes of starch-quercetin-α-amylase complex during molecular simulation


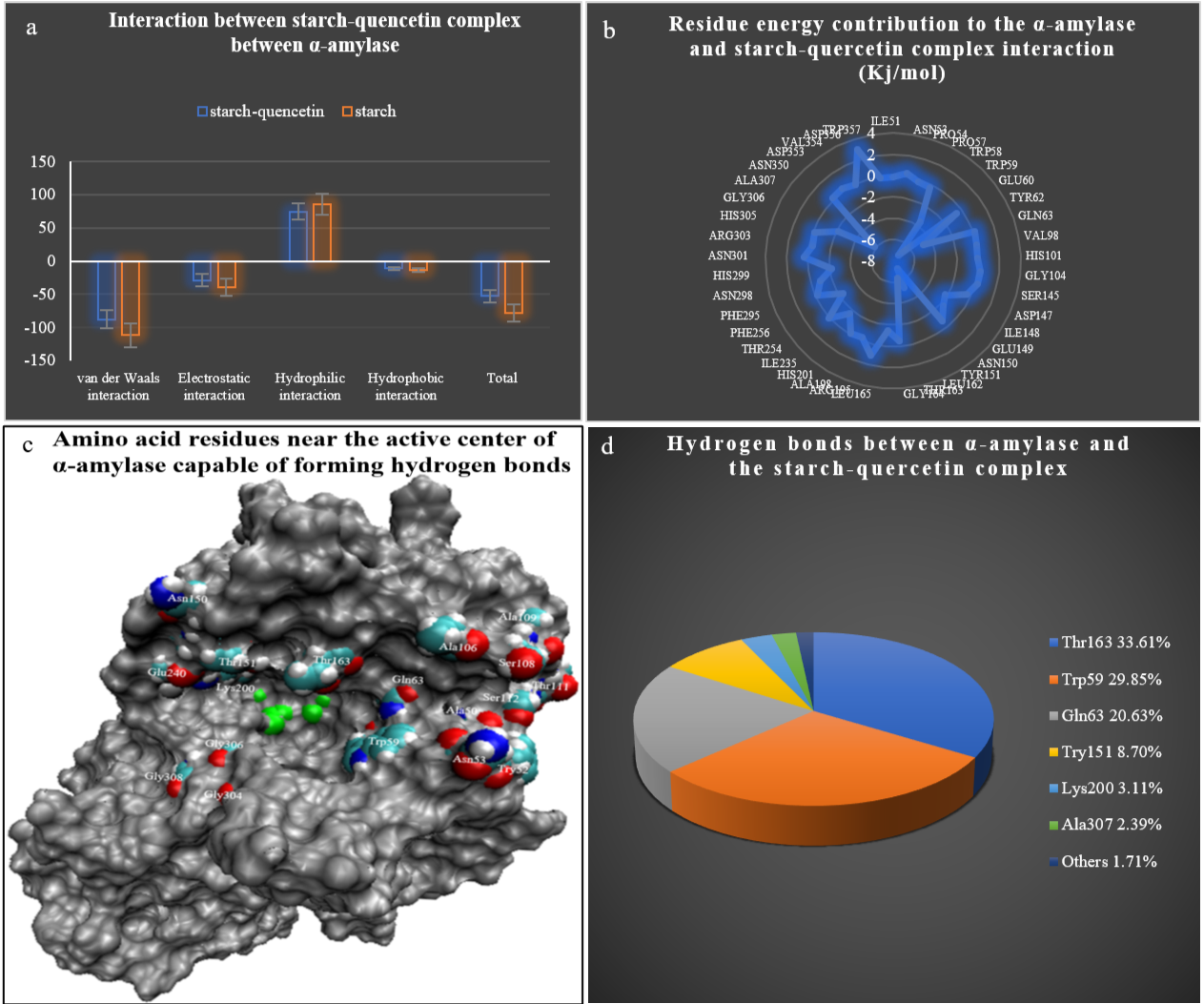


Fig. S11 Interaction forces between starch-quercetin complex and α-amylase
